# Oral administration of porcine epidemic diarrhea virus spike protein expressing in silkworm pupae failed to elicit immune responses in pigs

**DOI:** 10.1186/s13568-020-0952-9

**Published:** 2020-01-28

**Authors:** Chia-Yu Chang, Wei-Ting Hsu, Pei-Shiue Tsai, Chi-Min Chen, Ivan-Chen Cheng, Yu-Chan Chao, Hui-Wen Chang

**Affiliations:** 1 School of Veterinary Medicine, National Taiwan University, Taipei, 106 Taiwan; 2 Institute of Molecular Biology, Academia Sinica, Nankang, Taipei 115 Taiwan; 3 Graduate Institute of Life Sciences, National Defense Medical Center, Neihu, Taipei 114 Taiwan; 4 Chao-Kun Biotech Ltd, Taipei, Taiwan; 5 Graduate Institute of Molecular and Comparative Pathobiology, School of Veterinary Medicine, National Taiwan University, Taipei, 106 Taiwan

**Keywords:** Baculovirus expression vector system (BEVS), Porcine epidemic diarrhea virus (PEDV), Spike protein, Oral vaccine, Silkworm pupae (*Bombyx mori*)

## Abstract

The silkworm (*Bombyx mori*) and its pupae have been used for decades as nutritional additives and applied on the production of high-quality recombinant proteins via the baculovirus expression vector (BEV) system. The bio-capsule, the fat-rich body, and some body components of the silkworm pupae, which deliver antigens passing through the harsh environment of digestive tract and reaching the intestine, have been used as a vehicle for oral vaccines. In the present study, to develop a novel oral vaccine against porcine epidemic diarrhea virus (PEDV), the PEDV spike (S) protein was expressed in silkworm pupae and BmN cells using the BEV system. After three doses of oral administrations with 2-week intervals in pigs, neither PEDV S protein-specific humoral nor mucosal immune responses can be detected. The failure of eliciting the PEDV-specific immune response suggested that the BEV system using BmN cells or silkworm pupae as oral immunogen-expression vehicles was not able to overcome the immunological unresponsiveness, which was possibly due to gastrointestinal specific barriers and oral tolerance. Better strategies to enhance the delivery and immunogenicity of oral vaccines should be further investigated. Nevertheless, the PEDV S protein generated in the BmN cells and silkworm pupae herein provides an efficient tool to produce the recombinant antigen for future applications.

## Introduction

The baculovirus expression vector (BEV) system has been used as one of the most cost-effective and high productivity eukaryotic protein expression tools (Contreras-Gómez et al. 2014; van Oers et al. 2015). The baculovirus has a large, circular, double strained genomic DNA that is able to incorporate relatively long heterologous DNA insertions, which makes the baculovirus an excellent vehicle to express huge, complex proteins (Chambers et al. 2018). As a vector to express the recombinant proteins or to deliver foreign genes, the baculovirus enters cells use the viral glycoprotein 64 (GP64) to facilitate receptor binding endocytosis or low-pH-triggering membrane fusion (Hefferon et al. 1999; Zhou and Blissard 2008) and enter into different origins of cells. However, it only causes diseases in insects, especially the *Lepidoptera*, which makes the baculovirus a safe platform to produce recombinant proteins in vertebrates (Madhan et al. 2010; van Oers et al. 2015).

The BmNPV is an effective biovector to transduce recombinant genes into the silkworm (*Bombyx mori*) (Chambers et al. 2018), and can serve as a perfect bioreactor and a bio-factory to produce massive heterogeneous recombinant proteins (Rosales-Mendoza et al. 2016; van Oers et al. 2015). The capsule-like fat and the protease inhibitor of the silkworm prevent enzymatic digestion and the degradation of the recombinant proteins in the stomach when apply orally (Rosales-Mendoza et al. 2016). Besides, the pupae are regarded as one of the top-class unconventional protein source and had been utilized as the great energy food for the fish and poultry industry (Rangacharyulu et al. 2003). Comparing with the prokaryotic system, such as the *E. coli* or yeasts expressing system, recombinant proteins expressed by the baculovirus system undergo relatively more post-translational modifications, which is critical to induce appropriate immune response. Taking advantages from the high protein yield (generally 50- to 1000-fold higher than insect cell expression system), low cost, high nutrition, and high protein stability, the transgenic silkworm (*Bombyx mori*) has been applied to facilitate the development of the oral vaccines and the therapeutic protein drugs (Kato et al. 2010; Rosales-Mendoza et al. 2016). In human medicine, several cytokines or vaccines for the treatment of diabetes, hepatitis B virus, and *Helicobacter pylori* have been produced by the genetically engineered silkworm (Higashihashi et al. 1991; Rosales-Mendoza et al. 2016) in the veterinary medicine, the vaccine candidates for canine parvovirus, crap reovirus, crayfish white spot disease, and porcine foot-and-mouth disease virus derived from the silkworm have been demonstrated to stimulate both cellular and/or humoral immune responses, which were essential for the success of the vaccines (Feng et al. 2014; Li et al. 2012; Xu et al. 2006; Xue et al. 2013).

Porcine epidemic diarrhea (PED) is a severe infectious swine disease caused by porcine epidemic diarrhea virus (PEDV), which belongs to the family *Coronaviridae* and the genus Alphacoronavirus (Song and Park 2012). The PEDV can attack the intestinal villus epithelium of pigs and leads to the symptoms of watery diarrhea, vomiting, electrolyte imbalance, and even high mortality in suckling piglets (Jung and Saif 2015). New variants of PEDV which have high virulence had killed millions of neonatal piglets and brought about a 90–100% mortality rate that nearly destroyed the swine industry since 2010 (Song et al. 2015). The PEDV has an approximately 28 kilobases (kb), single strained, positive RNA as genome, it contains seven open reading frames (ORFs) encoding non-structural proteins and four structural proteins (Duarte et al. 1993; Jung and Saif 2015; Song and Park 2012). While the non-structural polyproteins are responsible for viral transcription and replication; the structure proteins, namely spike (S), envelope (E), membrane (M), and nucleocapsid (N) form the shape of the PEDV virions (Lee 2015). The S protein of PEDV can be separated further into S1 and S2 parts, and is in charge of the host-virus interaction and the establishment of the infection. Specifically, the S1 protein contains five conformational domains including domain 0, A, B, C, and D, which are in charge of the enteropathogenicity, receptor recognition, and viral neutralization (Li et al. 2017; Walls et al. 2016). The S2 protein is able to trigger viral internalization as well as being a target of viral neutralization (Okda et al. 2017). Due to above-mentioned crucial roles of the S protein to the PEDV, current development of vaccines against the PEDV is mainly based on the S protein (Song et al. 2015).

To develop a PEDV vaccine for providing both systemic and mucosal immunity, an oral vaccination strategy using a silkworm expression and delivery system to overcome the harsh PH environment and the digestion by the proteinase in the stomach (Silin et al. 2007) was used. To achieve this goal, the bacmid, pBPxhE-S-Bm, encoding the gene of recombinant full-length S protein of PEDV was constructed. After co-transfecting the pBPxhE-S-Bm with a BmNPV viral DNA, namely vBmpDsRFP, the recombinant baculovirus (S-Bm) was obtained. The expression of PEDV S protein in S-Bm inoculated cell line (BmN cells) and silkworm pupae were characterized, and the immunogenicity of PEDV S-expressing BmN cells as well as PEDV S-expressing silkworm pupae were evaluated in post-weaning pigs.

## Material and method

### Construction and the design of PEDV-S transfer bacmids

**Fig. 1:**
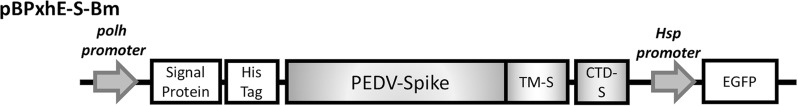
The construction map of the pBPxhE-S-Bm. The full-length S gene of PEDV were cloned into the pBPxhE plasmid and formed the pBPxhE-S-Bm in attempt to produce S protein anchored BmNPV. The original transmembrane domain (TM) and the cytoplasmic domain (CTD) of PEDV were retained for membrane anchoring. The expression of target gene was triggered by *polyhedrin (polh)* promoter which was followed by the signal peptide of GP64 and 6× His tag. In additionally, both constructed had an EGFP reporter that driven by the *heat shock protein* (*Hsp*) promoter

The full-length gene sequence of S protein of PEDV Pintung 52 strain passage five (PEDV-PT; GenBank Accession No. KY929405) were codon optimized (GenBank Accession No. MN586852) for the insect protein expression system and synthesized (ProTech, Taipei, Taiwan) as previously described (Chang et al. 2018a). In attempt to deliver the interest gene to the BmN cells, the gene of full-length S was cloned into pBPxhE transfer vector (pBPxhE-S-Bm), following the suggested protocol of the In-Fusion^®^ HD Cloning Kit (Clontech Laboratories Inc., Fremont, CA, USA) (Chang et al. 2012). The pBPxhE-S-Bm transfer vector contains a *polyhedrin (polh)* promoter of BmNPV, a viral GP64 signal peptide, and the 6× His tag that drive gene expression, lead protein synthesis, and label the target protein (Fig. 1). The plasmid also has an enhanced green fluorescent protein (EGFP) which driven by a *Drosophila heat*-*shock 70* (Hsp) promoter as a reporter fluorescence in the BmN cell and mammalian cells.

### Construction and viral titer determination of PEDV S displaying BmNPV

The recombinant BmNPV viral DNA, namely vBmpDsRFP, containing a Bsu36I restriction enzyme recognition site and tagged with a red fluorescent protein (RFP), was linearized by the digestion of Bsu36I restriction enzyme (NEB, Ipswich, MA, USA) under 37 °C for 1 h, then co-transfected with the PEDV-S transfer vector, the pBPxhE-S-Bm, to the BmN cells by using the TransIT^®^-Insect Transfection Reagent (Mirus, Madison, WI, USA). Five days after the transfection, to obtain the recombinant S-Bm virus, the cells were tenfold serial diluted in culture medium to perform the limited dilution for selecting a BmN cell exhibiting both EGFP (derived from pBPxhE-S-Bm) and REP (derived from vBmpDsRFP) positive. The viral titers of each clone were determined by the evaluations of 50% tissue culture infection dose per milliliter (TCID_50_/mL) (LaBarre and Lowy 2001). Briefly, the BmN cells (*Bombyx mori* ovary cell line, ATCC No. CRL-8910™) were cultured in TC 100 insect medium (Gibco, Gaithersburg, MD, USA) containing 10% fetal bovine serum (Gibco) at 26 °C and seeded in the 96 well plates with the total cell count of 4 × 10^4^. After 1 h incubation, each clone of the recombinant virus was serially diluted and applied onto the BmN cells with eight duplications. Following a 30-min centrifugation at 2000 rpm in order to promoting the viral infectivity, these plates were incubated at 26 °C for 4 to 5 days. The cytopathic effect (CPE) was observed and the viral titer of each clone were determined. The recombinant virus, namely S-Bm, which had a highest viral titer of 10^8^ TCID_50_/mL, was selected for the further characterizations and analysis. The S-Bm was propagated in the BmN cells and store at 4 °C until use.

### Detection of PEDV S expression in BmN cells by immunofluorescence assay (IFA)

The BmN cells in 80% confluency were inoculated with 10 multiplicity of infection (MOI) of the S-Bm and harvested 3 days after the inoculation. In order to detect the S protein expression in the BmN cells, the immunofluorescence assay (IFA) was performed. Briefly, the cells were fixed on the plates by using 4% paraformaldehyde (Sigma, MO, USA). After blocking with 3% BSA (Sigma, MO, USA) for 1 h and following by three times of PBS washes, the anti-PEDV S specific monoclonal antibody generated in our previous study (Chang et al. 2019), namely P4B-1, was applied on the cells for 1 h. Following three times washing with PBS, the goat anti-mouse IgG conjugated with Alexa Fluor 555 (Invitrogen, CA, USA) was applied onto the cells for 1 h. After washing with PBS, the cells were mounted with the Hoechst mounting solutions (Thermo Fisher Scientific, Waltham, MA, USA) to depict the nuclei. The result of florescence was observed under microscope.

### Expression and detection of the PEDV S in silkworm pupae

The pupae of the OJ03 × OJ04 strain *Bombyx mori* kindly provided by the Miaoli District Agricultural Research and Extension Station were directly injected with a total of 4 × 10^4^ TCID_50_ S-Bm in the volume of 100 μL. In the control group, the larva and the pupae were reared separately and injected with 4 × 10^4^ TCID_50_ wild-type BmNPV in the volume of 100 μL. Four days after the inoculation, the signal of EGFP fluorescence of the silkworm was observed under UV irradiation.

### Western blotting

To determine the protein expression level, the S-Bm infected BmN cells and the homogenized S-Bm infected pupae were collected. The BmN cells were collected 3 days after the S-Bm inoculation and lysed with the RIPA (Thermo Fisher Scientific, Waltham, MA, USA) buffer. Meanwhile, the S-Bm pupae were collected 4 days after inoculation. The hard puparium of pupae was removed, and the naked body of the pupae was homogenized by Bullet Blender Tissue Homogenizer (Next Advance Inc., NY, USA) with 1.5 mL lysis buffer containing 1 M Tris–HCl (Merck, Darmstadt, Germany), 0.5 M EDTA (Merck), 5 M NaCl (Merck), 10% (w/v) Brij96 (Merck), 10% (w/v) NP40 (Merck), 0.01% formalin (Merck) and sodium azide (Merck) (Chang et al. 2012). After centrifuge at 6000 rpm for 30 min, the supernatant of the homogenized sample was collected for the denaturation. The samples from the BmN cells and the homogenized pupae were denatured by boiling the samples under 95 °C for 10 min with the Laemmli Sample Buffer (Biotools, New Taipei city, Taiwan). The samples were then subjected to the electrophoresis in the gradient sodium dodecyl sulfate (SDS)-polyacrylamide electrophoresis (PAGE) gel (HR gradient gel solution, TOOLS, Taiwan) and then transferred to the PVDF membrane (Millipore, Darmstadt, Germany). The membrane containing protein samples was washed briefly in PBS (Omics bio, Taipei, Taiwan) and blocked by 5% skim milk for an hour at room temperature. Then, the mouse anti-6× His-tag monoclonal antibody (1:5000 dilution, EnoGene, NY, USA) was utilized as the primary antibody to probe the target proteins. Following by three times washing with PBS, the goat anti-mouse IgG antibody conjugated with HRP (1:5000 dilution, Invitrogen) was used as the secondary antibody for the signal detection. After three times washing, the signals were detected by using the Clarity™ Western ECL Blotting Substrates (Bio-Rad, CA, USA) using the Classic Blue Autoradiography film BX (Life Science, MO, USA).

### Immunization program of piglets

**Fig. 2:**
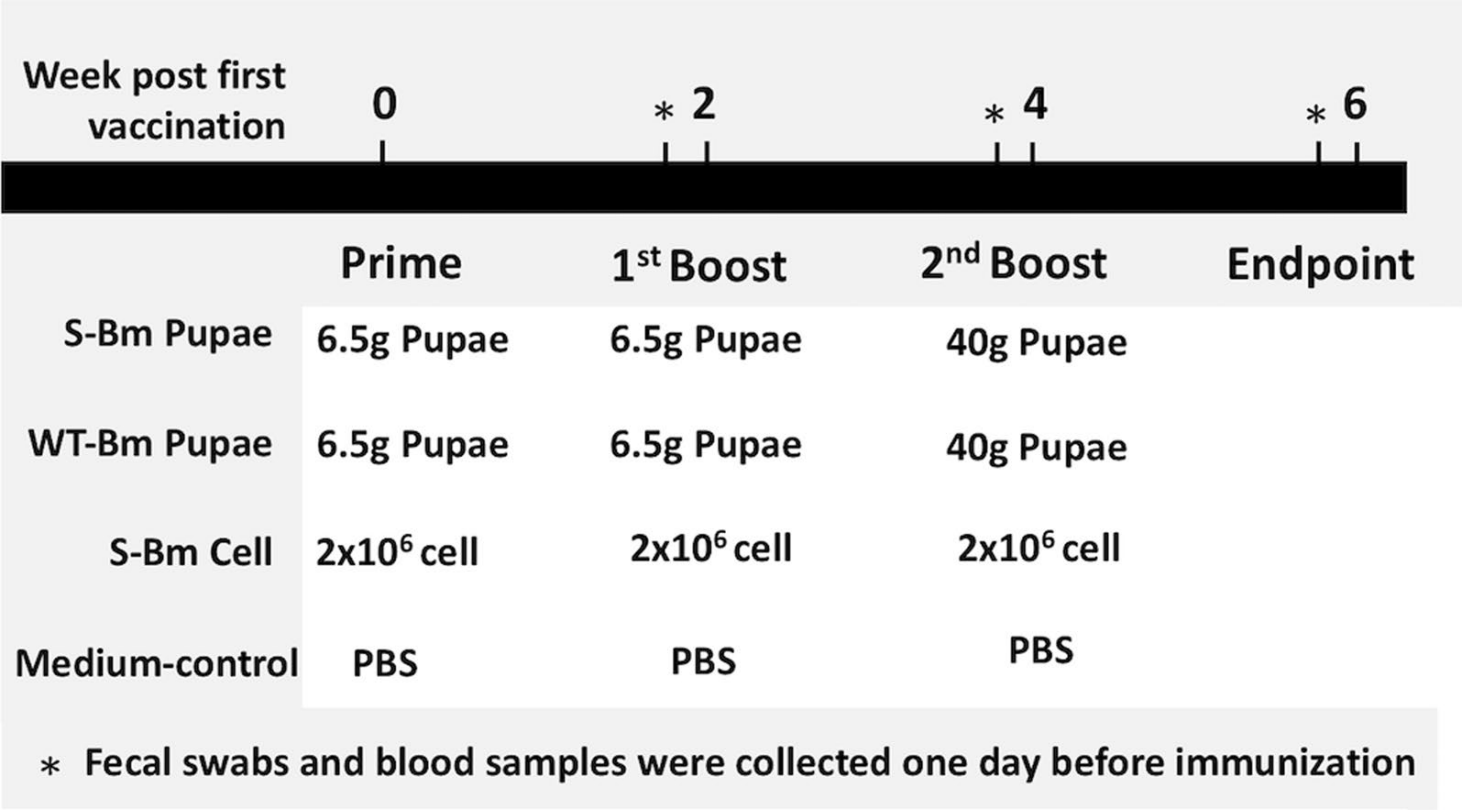
The oral immunization program of piglets. The S-Bm infected pupae or wild-type Bm infected pupae were orally administered to the piglets in different groups (namely ‘S-Bm Pupae’ or ‘WT-Bm Pupae’) at 0 week post first vaccination (WPFV), 2 WPFV, and 4 WPFV with a total of 6.5 g, 6.5 g, and 40 g of pupae. The S-Bm infected BmN cell were also orally administered to the piglets in the ‘S-Bm Cell’ group at 0 WPFV, 2 WPFV, and 4 WPFV with the total cell amount of 2 × 10^6^ per dosage. Orally inoculation of TC 100 to piglets (the ‘Medium-control’ group) was used as the negative control. *: At day 0, day 13, day 27, and day 41, blood samples and fecal swabs were collected for future analysis

The 4-week old, crossbred Large White × Duroc piglets were obtained from a conventional farm with no G2b PEDV outbreak history. Twenty piglets confirmed to be PEDV-seronegative by a PEDV S-specific ELISA were selected and used in this animal study. These piglets were separated into four groups according to the different treatments: the S-Bm cell group (n = 5), the control group (n = 5), the S-Bm pupae group (n = 5), and the WT-Bm pupae group (n = 5). As shown in Fig. 2, All animals were treated three times at 2 weeks interval. The piglets in the S-Bm cell group were orally fed with 2 × 10^6^ S-Bm infected BmN cells, which contained 12 μg recombinant S protein, in 5 mL TC 100 insect medium at 0, 2, and 4 week post first vaccination (WPFV); while the piglets in the control group were orally fed with 5 mL TC 100 insect medium. Each piglet in the S-Bm pupae and WT-Bm pupae group were orally feed with 6.5 g S-Bm-infected silkworm pupae containing 100 μg recombinant S protein or non-infected silkworm pupae at 0 and 2 WPFV, and fed with 40 g silkworm pupae containing 615 μg recombinant S protein at 4 WPFV. Piglets in different groups were housed in different rooms and all the pigs were labeled with ear tags. Ten milliliter blood samples in 1 mL 5% (w/v) EDTA anti-coagulated buffer and oral swabs were collected on day 0, day 13, day 27, and day 41 post priming for evaluating PEDV S-specific systemic IgG or mucosal IgA. The plasma of the blood samples was stored under − 20 °C until use. The oral swabs were re-suspended in 1 mL of PBS (Gibco, Gaithersburg, MD, USA) and also stored under − 20 °C until use.

### PEDV S-specific ELISA for detecting systemic IgG and oral mucosal IgA

The PEDV S-specific ELISA for detecting the systemic IgG and oral mucosal IgA of pigs was established and conducted as previous described with some modifications (Chang et al. 2018b). Briefly, the purified S protein of PEDV expressed by HEK 293 cells were diluted and coated on the Nunc maxi-soap plates (Thermo Fisher Scientific, Waltham, MA, USA) in the concentration of 2 ng/μL. After a 16-h incubation under 4 °C, the plates were washed six times with washing buffer (KPL, SeraCare, Milford, MA, USA) in the volume of 200 μL, then followed by an hour of blocking procedure by incubating the plates with blocking buffer (KPL, SeraCare) under room temperature. For detecting systemic PEDV specific IgG, plasma of piglets as well as a positive serum control and a negative serum control were 40-fold diluted in the blocking buffer (KPL, SeraCare) and applied onto the plates for an hour incubation. To detect the oral mucosal IgA level of piglets, the re-suspended supernatant of the oral swabs was twofold diluted in the blocking buffer (KPL, SeraCare) and applied onto the PEDV S coated plates for 16-h incubation under 4 °C. Then, the plates were washed three times with washing buffer and incubated with 1000-fold diluted secondary antibodies, the horseradish peroxidase (HRP)-conjugated goat anti-pig IgG (KPL, SeraCare) or a HRP-conjugated goat anti-pig IgA antibody (KPL, SeraCare) in the dilution of 5000-fold for another 1 h. After the plates were completely washed six times with wash buffer (KPL, SeraCare), the ABTS^®^ Peroxidase Substrates (KPL, SeraCare) was added onto the plates in the volume of 50 μL and incubated for 3 min under room temperature. To stop the coloration reaction, 50 μL stopping solution (KPL, SeraCare) was added. The plates were read by the EMax Plus Microplate Reader (Molecular Devices, Crawley, UK) under the wavelength of 405 nm to obtain the optical density (OD) values of each well. The value of positive control comes from a PEDV-hyperimmune pig, while the value of negative control comes from a seronegative piglet.

### Statistical analysis

The results of systemic IgG and IgA level were statistically analyzed by statistical analysis system version 9.4 (SAS 9.4, SAS Institute Inc., Cary, NC, USA) and compared by one-way analysis of variance (ANOVA). The significance was determined to have a *p* value < 0.05 (p < 0.05).

## Result

### S-Bm construction and PEDV S protein expression in BmN cells

**Fig. 3:**
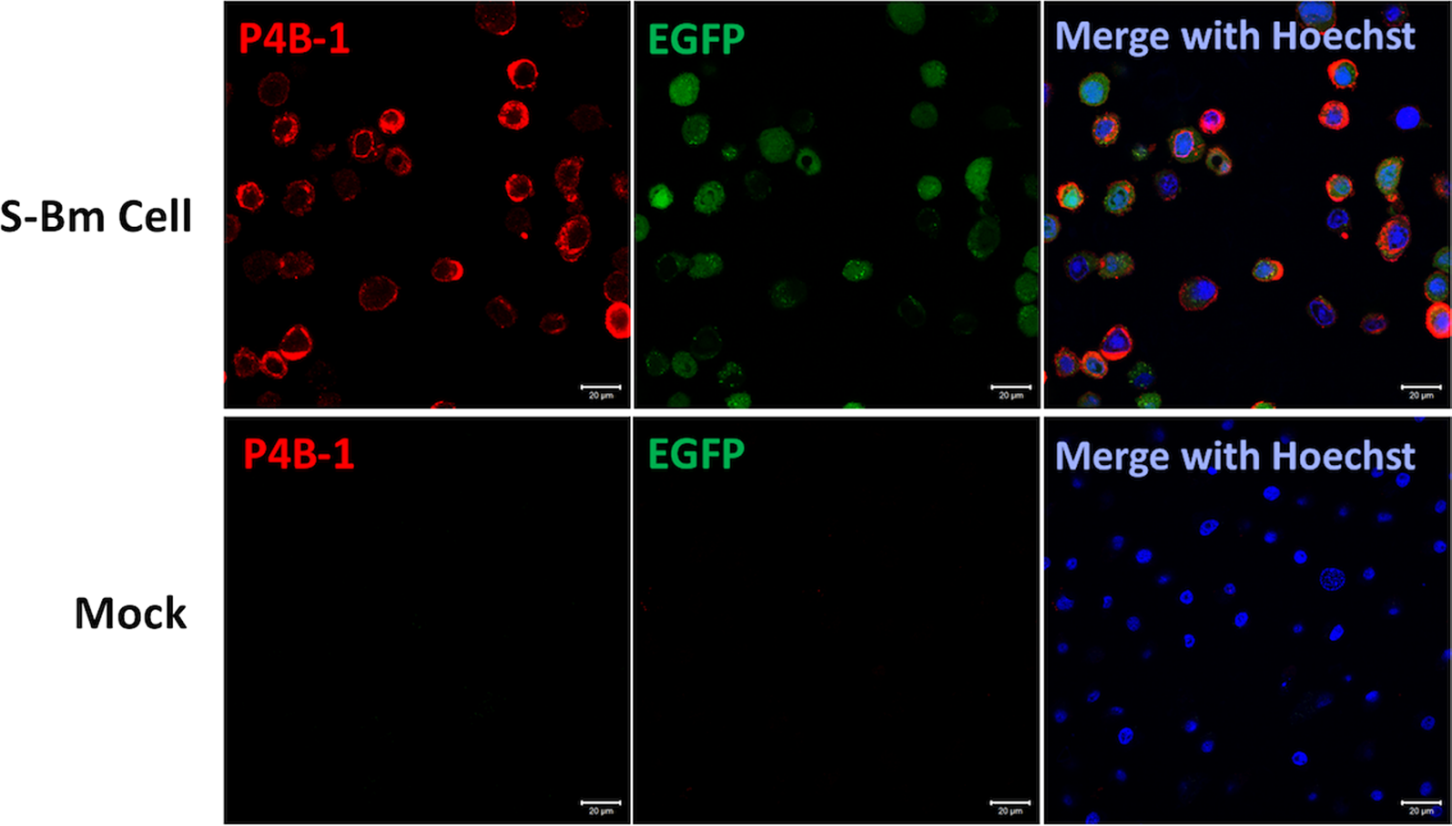
The immunofluorescence assay (IFA) of S-expressing BmN cells (S-Bm Cell). The BmN cells were inoculated with 10 multiplicity of infection (MOI) of the S-Bm and incubated for 48 h. The BmN cells without the inoculation were used as negative control (Mock). The cells were fixed with 4% paraformaldehyde and blocked with 3% BSA for 1 h. After washing with PBS, the S-expressing BmN cells (S-Bm cells) and the mock-inoculated BmN cells were probed with the anti-PEDV S monoclonal antibodies, P4B-1, for 1 h. Following the wash procedure with PBS, the goat anti-mouse IgG conjugated with Alexa Fluor 555 was used as secondary antibody. The Hoechst mounting solution was also used to demonstrate the nuclei. The fluorescence was observed under microscope

**Fig. 4:**
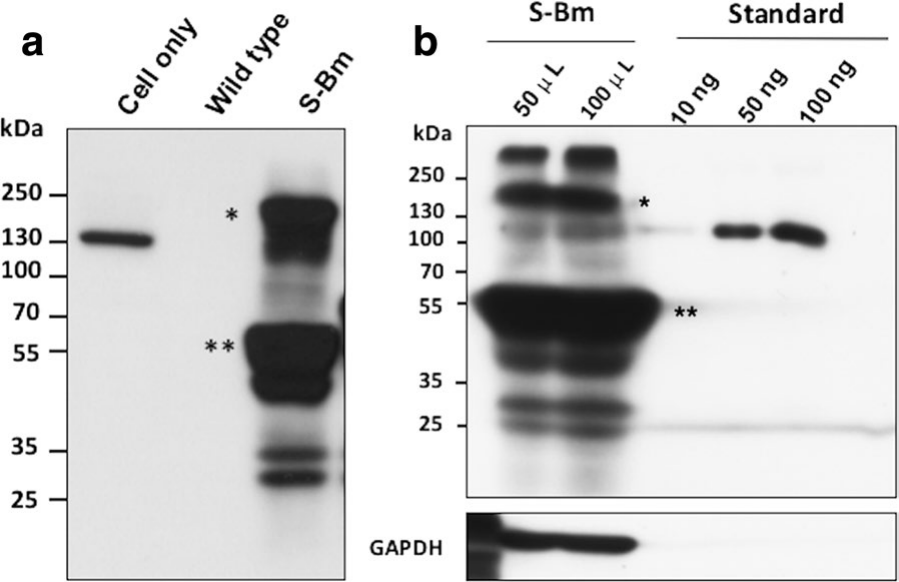
Detection of porcine epidemic diarrhea virus spike (PEDV S) protein expression level in the cell lysate of S-Bm inoculated BmN cells. The BmN cells inoculated with 10 multiplicity of infection (MOI) of the S-Bm were harvested and lysed 3 days after the inoculation. The proteins in the cellular lysate were separated by protein electrophoresis, transferred onto the PVDF membrane, and probed with the anti-6× His tag antibody. **a** The characterization of PEDV S protein from S-Bm inoculated BmN cells. Cell only: the samples from non-inoculated BmN cells; wild-type: the samples from wild type BmNPV inoculated BmN cells; *S-Bm* the samples from the S-Bm inoculated BmN cells. Single star icon represented the intact S protein on the predicted molecular weight; the double star icon represented the possible molecular weight of the cleaved S protein. **b** The determination of the yield of the PEDV S protein from S-Bm inoculated BmN cells. *S-Bm* the samples from the S-Bm inoculated BmN cells, *standard* the standard proteins of 6× His, *GAPDH* control cellular protein

Three days after co-inoculating the pBPxhE-S-Bm and the vBmpDsRFP bacmids in the BmN cells, the signals of the fluoresce of the EGFP and RFP were observed under the microscope. The double positive clones for the EGFP and RFP were picked and propagated in the BmN cells. The titers of each S-Bm viral clone were determined and the S-Bm clone which had a highest viral titer of 10^8^ TCID_50_/mL was selected. The expression of PEDV S protein in S-Bm infected BmN cells was detected by the IFA using anti-PEDV S-specific antibodies as well as western blotting using the anti-6× His tag antibody. In the Fig. 3, the recombinant S protein expressed on the S-Bm cells was successfully detected in more than 95% cells by using the anti-PEDV S monoclonal antibody, P4B-1. The fluorescence of EGFP was also observed on the S-Bm cells which indicated the successful transduction of S-Bm in BmN cells. As shown in Fig. 4, the size of the target protein migrated between 130 and 250 kilo dalton (kDa), corresponding to the predicted size of PEDV S protein (approximately 170–200 kDa). As referring to the standard proteins, the intact target S protein yield from 100 µL cellular lysate was higher than 100 ng.

### PEDV S protein expression in *Bombyx mori* pupae

**Fig. 5:**
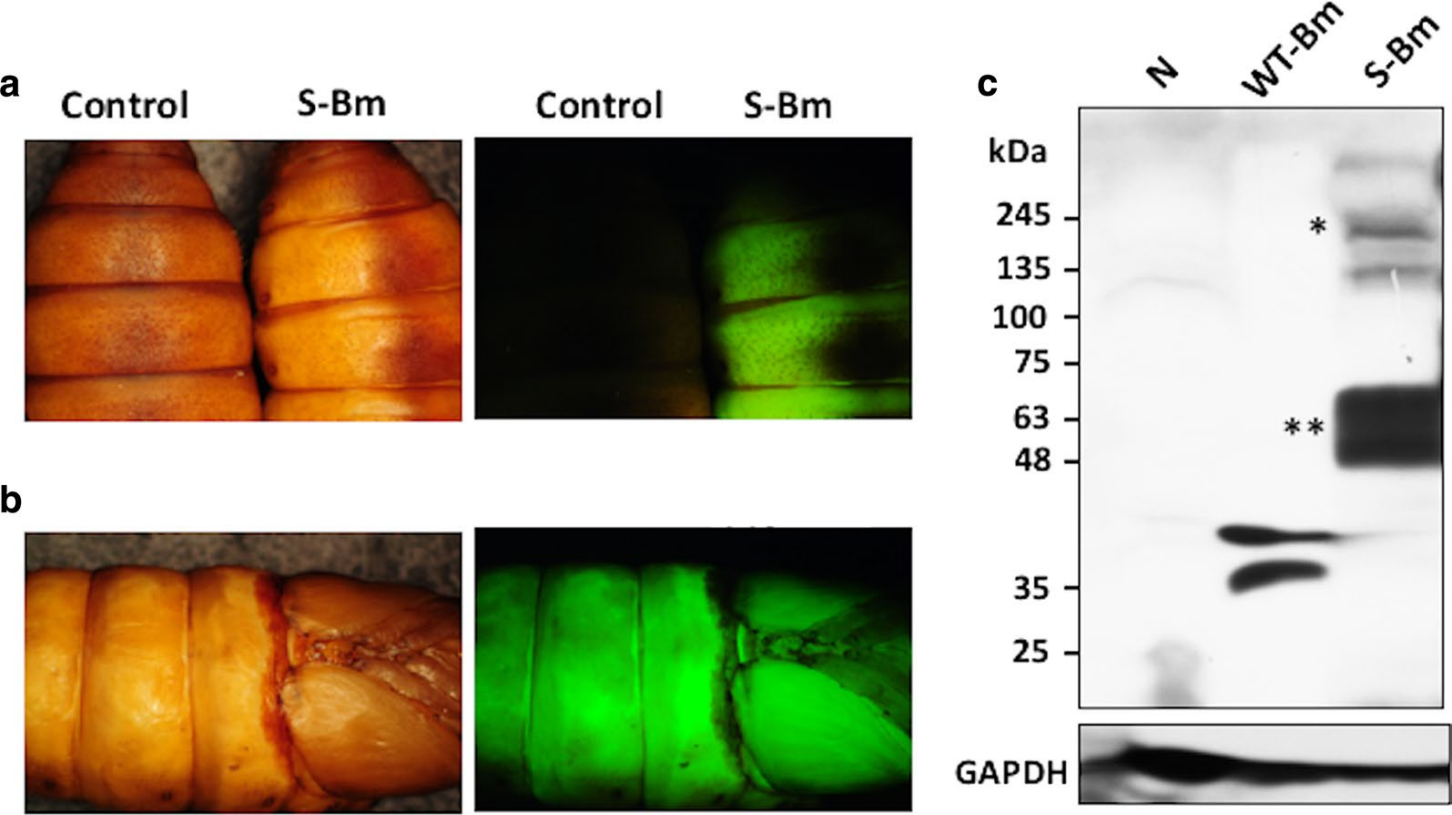
Detection of porcine epidemic diarrhea virus spike (PEDV S) protein expression in the S-Bm inoculated pupae. **a** The images of the pupae 3 days after the S-Bm inoculation under the visible light (left) or the UV emission (right). The EGFP signal of the S-Bm inoculated pupae was detected. Control: the pupae without S-Bm inoculation; S-Bm: the S-Bm inoculated pupae. **b** The distribution of the EGFP-conjugated recombinant S protein on the pupae. **c** The evaluation of recombinant S protein expression level of the pupae by the western blotting. The proteins from the homogenized body of the pupae were lysed, denatured, separated by protein electrophoresis, transferred onto the PVDF membrane, and probed with the anti-6× His tag antibody. The corresponding size of the S protein was predicted about 170–200 kDa. Single star icon represented the intact S protein on the predicted molecular weight; the double star icon represented the possible molecular weight of the cleaved S protein

After the injections of the S-Bm with 4 × 10^4^ TCID_50_, the homogenized tissue of the pupae was sampled for the western blotting which was probed with the anti-6× His tag antibody. As present in Fig. 5a, the pupae inoculated with S-Bm showed positive for the EGFP fluorescence under the UV emission, while no positive signal was present on the pupae in the non-inoculated control group. The distribution of the EGFP signal, which mean the distribution of the recombinant S protein, was included the whole body of the pupae (Fig. 5b). Moreover, the intact S protein of PEDV predicted as 170–200 kDa in molecular weight was detected from the homogenized tissue of the pupae by the western blotting (Fig. 5c). As referring to the standard proteins of 6× His, the intact target S protein yield was estimated at least 17 μg of recombinant S protein can be obtained from a pupa. However, a smaller protein, sized approximately 55 kDa, was also detected clearly by the western blotting, which indicated the incidence of the spontaneous cleavage of the S protein during the protein productive procedure.

### Systemic and mucosal PEDV S-specific Ig in piglets

**Fig. 6:**
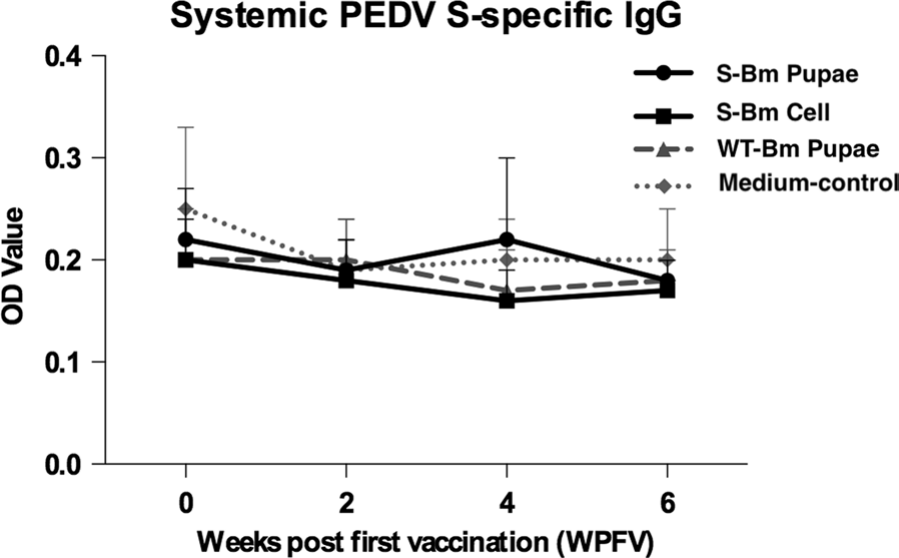
The evaluation of the systemic anti-porcine epidemic diarrhea (PEDV) spike (S)-specific IgG in piglets by the ELISA. Orally vaccinations were administered in 2-week intervals on week 0 (priming), week 2 (first boosting), and week 4 (second boosting). The blood samples were collected on week 0, week 2, week 4, and week 6. The data is shown as the means of optical density (OD) values with standard deviation (SD, the error bars). The solid line with round or square icons represented the trend of IgG level of the S-Bm pupae group or S-Bm cell group, respectively. The dotted line with triangle or diamond icons represented the trend of IgG level of the WT-Bm pupae or Medium-control groups. No significant difference with the control group (p < 0.05) were observed

**Fig. 7:**
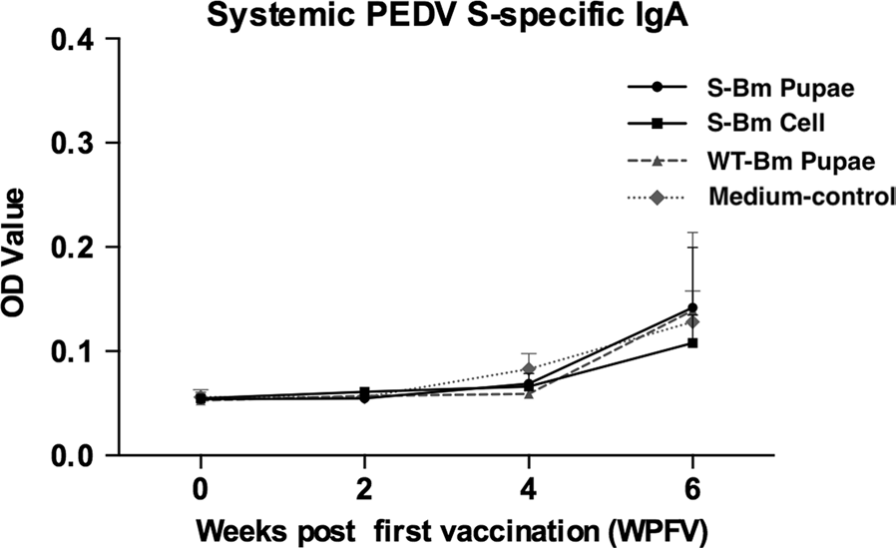
The evaluation of the oral anti-porcine epidemic diarrhea (PEDV) spike (S)-specific IgA in piglets by the ELISA. Orally vaccinations were administered in 2-week intervals on week 0 (priming), week 2 (first boosting), and week 4 (second boosting). The blood samples were collected on week 0, week 2, week 4, and week 6. The data is shown as the means of optical density (OD) values with standard deviation (SD, the error bars). The solid line with round or square icons represented the trend of IgG level of the S-Bm pupae group or S-Bm cell group, respectively. The dotted line with triangle or diamond icons represented the trend of IgG level of the WT-Bm pupae or Medium-control groups. No significant difference with the control group (p < 0.05) were observed

To evaluate the systemic immune response, the piglets were orally vaccinated with S-Bm pupae, S-Bm cells, WT-Bm pupae (wild type), or medium three times at 2-weeks intervals. The plasma PEDV S-specific IgG of each piglet was detected by ELISA at 0, 2, 4, and 6 WPFV. The optical density (OD) values of each plasma in different groups were analyzed and showed in mean ± standard deviation (SD). As present in Fig. 6, while the positive plasma from a hyperimmune serum used as the positive control of the ELISA had an OD value of 1.06, the PEDV-S specific IgG levels of all piglets orally immunized with S-Bm pupae or the S-Bm cells exhibited low OD values, < 0.35, which had no difference from those of the Medium-control and WT-Bm pupae groups. Similarity, after three times of oral administration, while the OD value of oral PEDV specific IgA level in the positive saliva sample was 0.74, the mean OD values of PEDV-S specific IgA levels in the WT-Bm pupae, Medium-control, S-Bm Pupae, and S-Bm cell groups were all under the OD value of 0.10 and had no significant difference (p < 0.05) among each other (Fig. 7).

## Discussion

Oral vaccination provides the possibility of stimulating both systemic and mucosal immune responses (Chattha et al. 2015), which might be a solution for development of an effective PEDV vaccine in pigs. In the present study, the S-Bm baculovirus has been successfully constructed, the full-length S protein of PEDV was also produced in both BmN cells and in the silkworm pupae. Although some cleaved S proteins were detected, both BmN cells and silkworm pupae expression systems were efficient to produce the full-length PEDV S protein. Taking advantages of the high productivity and the natural ability for the baculovirus to stimulate the immune responses, the S-expressing BmN cells and the pupae were subsequently applied as oral vaccines for PEDV. However, after three times oral vaccinations with either the S-expressing Bm N cells or pupae, no pigs showed detectable PEDV S specific seroconversion and mucosal IgA responses. The failure of eliciting the PEDV-specific humoral immune response suggested that the formula should be improved. Nevertheless, the high yield productivity of PEDV S protein in the S-Bm infected BmN cells and silkworm pupae provides an efficient tool to produce the recombinant antigen protein for future developments of PEDV vaccines and diagnostic tools.

Previously, several studies have achieved certain level of immunogenicity and protection against bacteria or viruses in mice, grass carp, or crayfish by directly oral administration of the recombinant proteins derived from silkworms (Wei and Xu 2009; Xu et al. 2006; Xue et al. 2013; Zhang et al. 2011). However, in contrast to these findings, oral administration of PEDV S protein expressing BmN cells or silkworm pupae was not able to elicit PEDV S specific immunogenicity in pigs in the present study. In fish and invertebrates, as compared with mammals, the less acidity of stomach and fore guts, and different enzymatic capabilities might allow more recombinant antigens with intact structural integrity passing through guts for stimulating oral immunity (Brown 2015). In the mice study, in addition to the UreB and HspA antigens of *Helicobacter pylori* were expressed in silkworm, a mucosal adjuvant, cholera toxin B subunit, was used to enhance oral immunity (Zhang et al. 2011). The high acidity and enzymatic capability in porcine stomach and intestine, and no adjuvant was added to overcome the oral tolerance might contribute to the failure of eliciting immunogenicity in pigs in our study.

To increase the efficacy of an oral vaccine, several improvements and key points could be addressed. First, the usage of mucosal-specific adjuvants is the most common strategy to enhance the mucosal immunity. For example, the cholera toxin subunits, cytokines, mucosal chemokines, and toll-like receptor ligands, chitosan…etc., are all popular choices for mucosal adjuvants (Rhee et al. 2012). These adjuvants were able to target the intestinal M-cells and enhance adhesion of the antigen or antigen carrying vectors to the mucosal epithelium or antigen presenting cells for facilitating the immunogenicity of the oral vaccine (Silin et al. 2007). In addition, encapsulating the antigen with PH-resistant and/or enzyme-resistant materials, such as poly (lactic-co-glycolic) acid (PLG), polysterene, polyethylene glycol (PEG), liposomes and cochleates…etc., may help to improve the integrity of the antigen and prevent its degradation in the gastrointestinal tract (Silin et al. 2007). The sizes of the capsule and the viscosity of the formula are also critical in developing the oral vaccines. For instance, an optimal size of approximately 10 μm particles would help to prolong contact of the antigen with villus mucosal membrane (Moser et al. 2003); a smaller size (approximately 0.2–0.5 μm) showed a better choice to be uptaken by the phagocytes and further antigen presentation (Moser et al. 2003). Some viral vectors, like the baculovirus, share the common receptors and broad tissue tropism thus are able to stimulate the immune responses in many hosts (Thiem and Cheng 2009).

In the present study, the yield of the intact recombinant S protein was estimated at least 17 μg per pupa and 6 μg in 1 × 10^6^ S-Bm infected BmN cells. However, large amounts of smaller protein, which was suspected as the cleaved S proteins, migrated to the molecular weight of 55–60 kDa was also detected. This observation indicates that the productivity of the S protein in BmN cells as well as the silkworm pupae was underestimated. The spontaneously cleavage or degradation of the recombinant proteins is often observed in the baculovirus protein expression system, especially using the bio-factory protein production methods such as the silkworm expression system (Kato et al. 2010). The possible reason is due to the spontaneous activation of baculovirus cysteine protease, v-cath, during the procedure of the protein production (Kadono-Okuda et al. 1995). To keep the integrity of the recombinant protein, a cysteine protease-deleted BmNPV might be used in the future for protein production by using the biofactory protein expression system of baculovirus.

In the present study, the absence of additive, mucosal-specific adjuvanted strategy might be responsible for the failure of inducing the immune reactions by oral administration of PEDV S-expressing pupae in piglets. Even though successfully eliciting the immunity by oral administrations with crude materials of antigen-expressing insect cells has been demonstrated in several studies (Wei and Xu 2009; Xu et al. 2006; Xue et al. 2013; Zhang et al. 2011), the differences of the microenvironment in the digestive tract and the discrepant activation mechanisms of immune system of different host species might contribute to the differences. Considering the immune tolerance and microenvironment of the digestive tract in pigs, the modification of formula and the usage of adjuvants to improve the stimulation of desirable and protective immune responses would be an important task for developing oral vaccines in pigs.

## Data Availability

The dataset(s) supporting the conclusions of this article is(are) included within the article (and its additional file(s))

## Abbreviations

BEV: baculovirus expression vector
PEDV: porcine epidemic diarrhea virus
S: spike protein
GP64: glycoprotein 64
PED: porcine epidemic diarrhea
ORFs: open reading frames
E: envelope
M: membrane
N: nucleocapsid
S-Bm: recombinant S-expressing baculovirus
PEDV-PT: PEDV Pintung 52 strain passage five
pBPxhE-S-Bm: pBPxhE transfer vector with the insertion of full-length S
EGFP: enhanced green fluorescent protein
Hsp: *Drosophila heat*-*shock 70*
TM: transmembrane domain
CTD: cytoplasmic domain
*Polh*: *polyhedron* promoter
*Hsp*: *heat shock protein*
RFP: red fluorescent protein
TCID_50_: 50% tissue culture infection dose
CPE: cytopathic effect
MOI: multiplicity of infection
HRP: horseradish peroxidase
OD: optical density
SD: standard deviation
